# Monitoring the Use of Human Milk, the Ideal Food for Very Low-Birth-Weight Infants—A Narrative Review

**DOI:** 10.3390/foods13050649

**Published:** 2024-02-21

**Authors:** Pasqua Anna Quitadamo, Federica Zambianco, Giuseppina Palumbo, Xavier Wagner, Maria Assunta Gentile, Antonio Mondelli

**Affiliations:** 1 Neonatal Intensive Care Unit, Casa Sollievo della Sofferenza, 71013 San Giovanni Rotondo, FG, Italy; palumbogiuseppina@tiscali.it (G.P.); xavierwagner5@hotmail.fr (X.W.); ma.gentile@operapadrepio.it (M.A.G.); a.mondelli@operapadrepio.it (A.M.); 2 Human Milk Bank, Casa Sollievo Della Sofferenza, 71013 San Giovanni Rotondo, FG, Italy; 3 San Raffaele Faculty of Medicine, University of San Raffaele Vita-Salute, 20132 Milan, MI, Italy; federicazambii@gmail.com; 4 Université Paris Cité, 79279 Paris, France

**Keywords:** very low birth weight, prematurity, mother’s own milk nutrition, human milk

## Abstract

Aware of the utmost importance of feeding premature babies—especially those of lower weight—with human milk, as well as the need to monitor this important element of neonatal care, we focused on four aspects in this review. First of all, we reviewed the beneficial effects of feeding premature infants with breast milk in the short and long term. Secondly, we performed a quantitative evaluation of the rates of breastfeeding and feeding with human milk in Very-Low-Birth-Weight infants (VLBWs) during hospitalization in the Neonatal Intensive Care Unit (NICU) and at discharge. Our aim was to take a snapshot of the current status of human milk-feeding care and track its trends over time. Then we analyzed, on the one hand, factors that have been proven to facilitate the use of maternal milk and, on the other hand, the risk factors of not feeding with breast milk. We also considered the spread of human milk banking so as to assess the availability of donated milk for the most vulnerable category of premature babies. Finally, we proposed a protocol designed as a tool for the systematic monitoring of actions that could be planned and implemented in NICUs in order to achieve the goal of feeding even more VLBWs with human milk.

## 1. Introduction

Taking into account the beneficial and sometimes salvific effects of breast milk for Very Low-Birth-Weight babies (birth weight < 1500 g), we evaluated whether data on the prevalence and duration of feeding with the mother’s own milk in the NICU and after discharge were available. We reviewed the information available on the determinants and predictors of nutrition with maternal milk: the data were insufficient and sometimes contradictory. Furthermore, we wondered whether the amount of donated milk that is available for this category of infants is known and how widespread human milk banking is, as we believe that they are elements that should be monitored globally. Only careful and systematic monitoring can result in an improvement in the feeding practices in NICUs and the effective promotion of breastfeeding. Common parameters should be identified to plan maternal milk feeding promotion and support actions in its favor, as such indicators could serve to guide measures that contain the risk of not feeding preterm infants with human milk. The ultimate goal is to understand how far away the goal of giving all premature babies the health opportunity represented by breast milk is. 

## 2. Maternal Milk Feeding and Outcomes in Very Low Birth Weight Infants

The survival rate of Very Low-Birth-Weight infants (VLBWs) has increased significantly in recent decades; thus, the attention of clinicians and researchers has focused on the neonatal complications affecting their prognosis and thus their both present and future quality of life. It has been shown that nutrition with breast milk is among the main factors in the prevention of prematurity-related comorbidities. 

Indeed, Mother’s Own Milk (MOM) feeding is vital for this population thanks to its ability to mitigate the permanent impact of preterm birth. The unique composition of MOM reduces the risk of prematurity-related diseases [1], such as necrotizing enterocolitis (NEC) [2,3,4,5,6,7,8,9], late-onset sepsis (LOS), bronchopulmonary dysplasia (BPD) [10,11,12,13], and retinopathy of prematurity (ROP) [14,15], but it also promotes better neurological outcomes [16]. Randomized Controlled Trials (RCTs) on breastfeeding have ethical implications for premature babies and, therefore, the only RCTs conducted have compared bank milk and formula [17,18]. 

In multicenter studies that have evaluated the effects of the policies of the implementation of exclusive feeding protocols with human milk, all results have pointed to a significant improvement in outcomes [5]. The clearest protective effect of breast milk is against NEC [2,3,4,5,6,7,8,9], and it persists even if donated human milk is used [8,19]. By contrast, a meta-analysis that included randomized controlled trials involving a total of 1879 preterm or low-birth-weight infants showed that artificial feeding led to a higher risk of NEC than feeding with donated human milk [20].

We can say that for the prevention of NEC, breast milk is considered a kind of drug with dose-dependent effects; moreover, a plethora of scientific studies have highlighted a significant reduction in the incidence of NEC thanks to feeding with breast milk. These results are in line with another of the most important beneficial effects of Human Milk (HM), namely better food tolerance [21,22,23], resulting in the faster achievement of full enteral feeding. This is linked to the multiple bioactive elements of HM, in particular oligosaccharides, but it is also due to the different gut microbiota of premature babies fed with breast milk, which is richer and more harmonious; this is another fundamental element for their present and future well-being [4,24].

A multicenter cohort study [10] with a sample of 1433 VLBWs showed that the use of breast milk was linked to a reduced risk of BPD, as well as NEC and ROP. The effect on bronchopulmonary dysplasia was consistent with that found in an observational study [12] on 926 VLBWs that showed an association between feeding very preterm infants with fresh milk expressed by the mother and a lower incidence of BPD. 

As with a therapeutic resource, the impact on reducing life-threatening risks and complications associated with prematurity, as well as the effects on neurodevelopment and health after discharge with less recourse to rehospitalizations, is related to the volume and duration of exposure to MOM [21,25].

Some authors have described the effects of HM at all volumes administered, whereas in other studies, the daily “doses” are indicated. An update in 2016 [15] reported a strong association between any amount of HM and protection against all forms of ROP, even the most severe one. In a large study conducted by Meinzen-Derr et al. [25] on 1272 ELBWs, a 13% reduction in the risk of NEC or death was demonstrated with a dose of 100 mL/kg of human milk administered in the first 14 days of life. In a systematic review [26], a reduction in the incidence of NEC, LOS, and ExtraUterin Growth Retardation (EUGR) in VLBWs was observed with a dose of at least 50 mL/kg of HM per day for the first 4 weeks. 

In a recent retrospective analysis [27] of 630 VLBWs, MOM feeding within 72 h of birth and the persistence of breast milk feeding during hospitalization were able to reduce the incidence of moderate and severe BPD.

This is particularly relevant considering that BPD is an increasingly common respiratory adverse outcome that prolongs NICU stays and particularly impacts rehospitalization, as well as long-term lung and neurological outcomes. In a study conducted on rats [28], the role of HBM-Exo in the protective action of breast milk on BPD was investigated, and the results were also encouraging about the possibility of a new therapeutic approach to BPD. HBM-Exo histochemically promoted the proliferation of AT II cells and inhibited their apoptosis through IL-17 regulation, and in the overall picture, it improved the appearance of structural lesions in the lung tissue. In this regard, it should be remembered that, despite the existing awareness of the richness of bio-factors and the cellular heritage of breast milk, little is known yet about the mechanisms underlying their protective and, in some cases, curative effects. Therefore, there are wide margins for study and research, as well as possible future clinical applications. 

Research on human milk is rapidly expanding and modern technologies may be of help, for example, in identifying the mechanisms underlying the beneficial effects of human milk on neurodevelopment. There is an important demand for insights into the neuroprotective or neuroreparative potential of specific bioactive components of milk. Additionally, emerging studies support the effects of human milk on structural brain development, such as increased white matter development and increased cortical thickness. 

In a multicenter study [29] evaluating the neurodevelopmental assessment of 252 Extremely Low-Birth-Weight (ELBW) infants (<1250 g), those fed with HM had significantly higher cognitive scores at 18–22 months of corrected age. It should be marked that brain MRI, performed when the premature baby comes to term, is increasingly used to study the effects of human milk on the preterm brain. In a review [30] that selected and evaluated seven observational studies carried out between 2013 and 2021, the most significant element arising from the associations observed was the finding of regional and tissue-specific neuroprotective effects on known areas of vulnerability in the preterm infant. In addition, greater exposure to human milk rather than formula was associated with a more mature white matter with fewer lesions and greater regional brain volumes, particularly in deep nuclear gray matter, the amygdala–hippocampus, and cerebellum.

In a review [31] in which particular attention was paid to myelination and epigenetic modifications, several elements (milk fat globule membrane, lactoferrin, HMOs, microbes, osteopontin, milk exosomes) were identified for their potential action on brain development through various possible mechanisms. 

The more studied breast milk components that mediate improved cognitive outcomes [32,33] include long-chain polyunsaturated fatty acids and human milk oligosaccharides. 

Another of the most recent and interesting areas of research concerns the cardiovascular health of premature infants. Indeed, the cardiac phenotype of young adults born prematurely results from early postnatal cardiac remodeling induced by prematurity and associated pathologies. It is described [34] as a reduced biventricular volume, a relatively lower systolic and diastolic function, and a disproportionate increase in muscle mass. The clinical counterpart of this phenotype would be a reduced exercise tolerance and an increased risk of hypertension and cardiovascular events. Recent studies [35] suggest that early exposure to MOM can mitigate the remote cardiovascular outcomes of premature birth, by slowing down or even stopping these pathophysiological changes.

It should also be remembered that improving the health outcomes of preterm infants can lead to significant reductions in neonatal intensive care costs, but also those of the short and long-term care of children and those of society as a whole. The average cost of care for the first 6 months of life for a full-term baby was estimated by Johnson et al. [36] to be USD 7247 (USD 2019), well below the USD 332,225 estimated for a VLBW preterm baby.

Clinical studies have shown that not only the health benefits, but also the cost savings associated with MOM, are often dose dependent [37].

Data [38] from the LOVE (Longitudinal Outcomes of VLBW Infants Exposed to Mothers’ Own Milk) cohort study on 430 VLBW children enrolled between 2008 and 2012 revealed a dose–response relationship [39] between the increased amounts of MOM received at critical periods of exposure during stay in an NICU and the protective effects, not only on the risk of NEC, sepsis and BPD, but also on their cost reduction. This dose–response association was also observed with higher cognitive index scores at 20 months in VLBW children receiving higher amounts of MOM. 

The Trisha Johnson’s group found and reported that, in VLBW babies, the costs of hospitalization in the NICU attributed to complications that could potentially have been prevented if MOM had been used are high, and that each complication increases them by 15–30%. More precisely, the cost savings attributed to a reduction in the incidence of these complications were estimated as being within a range from USD 27,790 to USD 46,103 [36]. The authors state that, considering that the care of VLBWs is among the most expensive in a hospital, the results of the study could represent a stimulus for policymakers, taxpayers and health professionals to invest more in optimizing VLBW nutrition with a more efficient and cost-effective strategy.

## 3. Prevalence and Duration of Premature Babies’ MOM Feeding 

Despite this, studies have shown that far fewer preterm infants are breastfed exclusively at discharge than full-term ones: 45% of preterm infants in Sweden [27] and 68% in Denmark [40] compared to 75% of 10-day-old full-term infants [41]. While this observation is comparable to that reported in an Australian study showing that 55% of preterm infants are exclusively breastfed [42], this proportion is even smaller in other countries, with 23% of them being exclusively breastfed in a Japanese study [43], 22% in an US study [44], and 20% in a European study conducted in 11 countries [45].

The general picture (Table 1) that emerges from the unsystematic published works on the subject does not appear encouraging, because performances fall far short of WHO standards. However, in some states, promotion policies have begun to positively influence the feeding of premature babies with maternal milk. In fact, in recent decades, breastfeeding rates have increased substantially, but the variability between NICUs and countries is huge. For example, in France, the breastfeeding rates increased from 19% in 1997 to 47% in 2011 [46].

In Germany, a study [47] reported that out of 368 preterm infants, 60.1% were discharged with exclusive MOM feeding; this is far more than the 25% of exclusive and the 47% of at least partial MOM feeding rates at discharge in children under 32 weeks Gestational Age (GA), as shown by the EPIPAGE-2 cohort study [46].

A low incidence of breastfeeding was recorded in Greece, as in other Southern European countries, such as Portugal [48]. Although these breastfeeding rates were low and unsatisfactory, they have improved over time. In fact, these rates have increased during NICU hospitalization (44%), at discharge (48%) and during the first days at home (46%), compared to data from ten years ago [49]. The same messages of hope come from a national study on the frequency and determinants of breastfeeding in Greece, published by the Institute of Child Health and the National School of Public Health [50]. The authors report a significant increase in early breastfeeding rates, with 53.6% of infants exclusively or partially breastfed in NICUs; however, at the end of the first month after birth, only 23.5% of premature babies were exclusively breastfed. Comparing the same European regions in the MOSAIC [51] (2003) and in the EPICE (2011/2012) studies [46], the prevalence of at least partial and exclusive MOM feeding at discharge improved in all regions over time, with a few exceptions (Lazio (Italy) and Wielkopolska (Poland)). In Portugal, there was a substantial increase in any MOM and exclusive MOM feeding rates at discharge from 2003 to 2011/2012. Another Portuguese study from 2013/2014 [52], which included all level III NICUs in the Northern region, provides a brilliant example by reporting a 96.7% prevalence of breast milk supply in families of VLBWs at 2–3 weeks of life. 

In Northern European countries, such as Denmark [53,54], the incidence of breastfeeding is historically high in premature infants. Exclusive MOM feeding at discharge varied among NICUs from 53% to 83% in a Danish prospective cohort of preterm infants. However, something has changed in Sweden, a country with excellent performances and results obtained in terms of feeding newborns with breast milk [55,56,57,58]. Swedish data from the last 10 years show a significant reduction in the rates of exclusive breastfeeding in preterm infants, especially in ELBWs (Extremely Low Birth Weight infants), with a decrease from 55% to 16% in neonates born between 22 and 27 weeks GA, from 41% to 34% in babies born between 28 and 31 weeks GA, and from 64% to 49% in moderately preterm infants (GA 32–36 weeks) [27]. 

In the California Perinatal Quality Care collaborative cohort [59] of 6790 VLBW infants born in 2005 and 2006, breastfeeding rates ranged widely from 19.7% to 100%, with an average of 61.1% at NICU discharge. 

More recently, it was reported that in the United States, 75% of preterm infants in NICUs were breastfed in 2015 [60]. Then, at the Boston Medical Center in early 2015, the rate of breast milk initiation among infants at <34 weeks of gestation was >80%, but <60% of them continued breastfeeding until the time of discharge [61]. In a population-based cohort study [62] of 6404 children born at <33 weeks GA included in a Collaborative National Network between 2015 and 2018, 4457 (70%) received exclusive MOM or MOM supplemented with formula. MOM feeding rates at discharge ranged from 49% to 87% among NICUs. 

National data [63] from more than 800 NICUs of the Vermont Oxford Network Quality Collaborative showed that human milk feeding at discharge among VLBW infants increased from 44% in 2008 to 52% in 2017.

Data from the Centers for Disease Control and Prevention [64] indicate that the rates of breast milk received among extremely preterm, preterm, late preterm and full-term infants were 71.3%, 76.0%, 77.3%, and 84.6%, respectively, among babies born in 48 states and in the District of Columbia in 2017. 

In Brazil [65], the prevalence of exclusive breastfeeding for VLBWs was 65.2% at discharge, 51% at 3 months, and 20.6% at 6 months. 

Large regional differences in the breastfeeding rates of preterm infants at discharge and in the following months exist between European countries [51,66]. 

In the MOSAIC study [51] conducted in 2003 in eight European regions on a cohort of 3006 babies born before 32 weeks GA and discharged home from the NICU, breastfeeding rates ranged from 19% in Burgundy, France, to 70% in the Lazio Region in Italy. Variations between regions and neonatal units remained statistically significant after adjustment for maternal, infant and unit characteristics.

Results from the EPICE [67] cohort, providing data from 19 European regions (6592 infants), showed that exclusive MOM feeding was provided to 37.5% of infants in the first 24 h after the first enteral feeding. At discharge, 58% of infants received any MOM, whereas 27.5% were exclusively fed with MOM, and a wide variation between regions and within countries was confirmed. The Eastern region of Denmark presented the highest percentage (80%) of infants fed with any MOM at discharge, and the Northern region of the UK had the lowest proportion (36%). The East-central region of the Netherlands had the main proportion of exclusive MOM at discharge, i.e., 51.5%. In Portugal [68], 65% of infants received exclusive mother’s own milk within the first 24 h after the first enteral feeding, nearly two-thirds of infants received some breast milk at discharge, and 25% received it exclusively. Among infants fed with any MOM at discharge, 68% were breastfed, with a wide range between 16% and 93% among the regions. 

In an Italian survey [69], 45% of infants with a birth weight <1500 g and 23% of infants over 2500 g did not receive MOM at discharge. 

In a more recent study [70] that enrolled 64 mothers and 81 children, Gianni et al. reported breastfeeding in any proportion in 66% of preterm infants at discharge, 27% of whom were exclusively breastfed. Our NICU practices [71] have improved over the years, with MOM feeding rates in the NICU increasing from 66.67% to 76.47%, and the percentage of MOM feeding at discharge increasing from 45.8% in 2017 (Exclusive MOM 33.3%) to 58.82% (Exclusive MOM 41.18%) in 2021.

In China, there is a high variability between hospitals in the prevalence of breastfeeding preterm infants [72]. In 2017, Hei et al. [73] found a high breastfeeding rate (breast milk > 1/2 of the daily feeding volume) of 55.78% in a population of infants between 28 and 35 weeks GA from 11 NICUs. In 2018, another Chinese study [74] involving 113 preterm infants (<34 weeks GA) reported breastfeeding and exclusive breastfeeding rates of 58.2% and 18.8%, respectively. However, in China, although the breastfeeding of preterm infants has improved in recent years, the separation of the mother–child dyad in NICUs [75] is one of the main factors acting as an obstacle to feeding with MOM.

In the literature [56], we found few studies about feeding VLBWs after discharge. We can report data from two Portuguese regions [68] that describe high percentages of partial breastfeeding, namely 91.0% of babies <32 weeks GA and 65.3% of exclusive mother milk feeding; however, there is a less interesting duration, with a median value of 2 months for exclusive MOM and 3 months for mixed MOM feeding. Only 9.9% of these premature babies received exclusive MOM for at least 6 months, 10.2% received mixed MOM for 12 months or more, and 2.0% received MOM for up to 24 months. Also in the current Portuguese real-life context, other studies [68] reported a low prevalence of exclusive breastfeeding at 6 months (from 1.0% to 27.0%) and mixed MOM feeding at 12 months (from 8.0% to 12.0%).

In a study conducted in Greece [76], 78.1% of preterm infants received MOM during the first days of life and 58.1% were exclusively breastfed during the first month. The trend recorded over the following months was a gradual decrease in breastfeeding to 36.9% in the third month and 19.4% in the sixth month. The prevalence of breastfed infants was 14.7% at twelve months and 7.5% at eighteen months. Data on the prevalence and duration of MOM feeding are summarized in the table below (Table 1).

**Table 1:** Feeding VLBW infants with MOM in NICU, at discharge and post-discharge.

	Sample	In NICU	At Discharge	Post-Discharge	References
Australia	735		55%		2008 Dodrill [42]
Japan	115		22.6%	15.7% after 5 months	2011 Mamemoto [43]
Sweden	2751		93%	36% after 6 months	2003 Flacking [58]
Swedish Neonatal Register	29,445		Exclusive		2016 Ericson [27]
	GA 22–27 w		55% 2004		
			16% 2013		
	GA 28–31 w		41% 2004		
			34% 2013		
	GA 32–36 w		64% 2004		
			49% 2013		
Germany	368		60.1 exclusive		2021 Heller [47]
	8222				
Portugal		12%	17%	24 first days at home	
		44%	48%	46%	
	580		25.2% exclusive	9.9% after 6 months	2018 Rodrigues [48]
			40.7% mixed	10.2% mixed after 12 months 2% mixed after 24 months	
			65.3% exclusive		
			91% mixed		
Denmark	1488		68% exclusive		2014 Maastrupt [54]
			17% mixed		
France	7804		47.2% (21.1–84%)		2019 EPIPAGE cohort Mitha [46]
Greece	100	12% exclusive 44% mixed	17% 48% mixed	24% first days 46% first days	2020 Daglas [54]
	279	78.2% start	58.1% 1st month	36.9% after 3 months 19.4% after 6 months 14.7% after 12 months 7.5% after 18 months	2022 Sokou [76]
		53.6% mixed	23.5% mixed		
Italy	2948	55% VLBW	31% VLBW		2013 D’avanzo [69]
			66% mixed 27% exclusive		2018 Gianni [70]
Europe	3006		Lazio -70% mixed -18% exclusive Trent region UK -35% mixed -29% exclusive Île-de-France region -24% mixed -14% exclusive		2003 Bonet [51] MOSAIC cohort
	6592	46.5%	58% (36–80%)		2018 Wilson [67]
USA					
		68% start 52% during stay	48%		2010 Pineda [44]
California Perinatal Quality Care Collaborative	6790		61.1% (19.7–100%) (exclusive + mixed)		2009 Lee [59]
BMC		94% start	47%		2019 Kalluri [66]
CNN	6404 2015–2018		70% (49–87%)		2021 Dharel [62]
Vermont Oxford Network			44% (2008) 52% (2017)		2019 Parker [63]
CDC			71.3%		2017 Chiang [64]

## 4. Determinants and Predictors of VLBWs’ Nutrition with Maternal Milk 

The low prevalence and short duration of MOM feeding among VLBW infants have been associated with several factors [77]. 

Several studies have shown that mothers who are not married, younger, have a lower educational level, are smokers, multiparous and do not attend prenatal care are less likely to feed their infants with MOM [51,78,79,80]. Furthermore, infants with a lower gestational age, lower birth weight, severe neonatal morbidities and longer hospital stays are less likely to be fed with breast milk [78,79]. The European EPICE cohort has also explored the maternal, obstetric and infant factors, as well as the maternal and neonatal unit policies that may influence MOM feeding at hospital discharge [67]. Vaginal delivery, singleton delivery and MOM consumption at the first meal have been associated with exclusive MOM feeding, while a positive association with any MOM feeding at discharge is described for factors such as the administration of prenatal corticosteroids, primiparity, timing < 24 h after birth before the first enteral feeding and MOM consumption at the first meal. Additionally, this study demonstrated that units with a Baby-Friendly Hospital accreditation had improved MOM feeding rates at discharge. On the other hand, units with protocols on the use of MOM and units using donor milk had higher rates of exclusive MOM feeding. 

The current literature suggests, with extensive evidence, that the degree of promotion and support by NICU staff, as well as the unit’s policies and practices, impact MOM feeding rates among preterm infants [78,80,81,82,83]. The results from the EPIPAGE cohort also revealed that MOM feeding at discharge, after adjustment for individual variables, was associated with kangaroo care provision at any time during the first week of life, and with policies supporting feeding with MOM [51]. These include the systematic information of mothers hospitalized for the threat of premature birth and the proposal of breast milk expression within six hours of birth, in the presence of a special room for this expression and the availability of dedicated protocols. 

To better understand this aspect, we delineated the factors into two groups, with or without the possibility of modification with targeted actions. Let us start by saying that the data are often contradictory, but an accurate analysis could give an idea of how these elements can impact the goal of breastfeeding premature babies, particularly VLBWs.

### 4.1. Non-Modifiable Factors

A young maternal age is often considered a risk factor for the lack or absence of MOM feeding at discharge. In a population of preterm infants <34 weeks GA, each year of maternal age was associated with a 1.24-fold increase in breastfeeding at discharge [84]. In another report [66], mothers aged under 25 years stopped breastfeeding more often before discharge and before six months than mothers older than 25 years. This age limit is frequently indicated; in fact, in a further study, infants with mothers younger than 25 were 30% less likely to be breastfed than children with older mothers [51]. 

The EPICE Research Group, in a logistic analysis, found an association between exclusive breastfeeding at discharge and two factors, which are the young age of the mother and the early onset of oral nutrition [45]. This association has also been found in an Italian survey [85]. 

Gestational age is also one of the most considered elements. For example, preterm babies < 28 weeks GA had a 2.9 times greater risk of exclusive breastfeeding failure [53]. In our previous publication [86] focused on the amount of donated milk, the data analysis revealed that the maternal age, profession of the donors, and birth weight of their children had a statistically significant impact on the Donor Milk (DM) volume, while the gestational age of the donors’ children influenced the milk donation volume; however, this lacked statistical validity.

Data on multiple births are controversial because some studies show an association with exclusive breastfeeding [44], while others [53,78,84] show a correlation with Formulated Milk (FM) feeding or the more frequent discontinuation of breastfeeding before six months of age. 

In our NICU [87], the percentage of twins almost doubled between 2015 and 2020. In total, 18.1% of twins received breast milk for more than 6 months and 6.3% for more than 12 months. It is worth reporting that twins of lower gestational age and weight, born to multiparous, more mature and educated mothers, received breast milk for a longer period.

In another study [62], the MOM feeding rates at discharge were associated with a higher gestational age at birth, with better outcomes among those born at 29–32 weeks GA compared to those <26 weeks GA. Other determinants associated with a poorer MOM feeding rate at discharge were birth to primiparous mothers or birth to mothers with diabetes. 

In studies [88] conducted in the United States, the mother’s marital status is often assessed, and the results are not univocal. In full-term infants, married women started and maintained breastfeeding longer than single mothers [89], while in another report [44], unmarried women started feeding premature babies with MOM more often and more often provided breast milk until discharge. In another study [90], children of married mothers were discharged more frequently with MOM feeding. European studies rarely mention the mother’s marital status; thus, no data are available in this regard.

The socioeconomic status of the mother influences breastfeeding in both full-term and preterm infants: mothers with a lower level of education or with less access to care stop breastfeeding earlier at discharge [54] and before six months [91]. In European countries [84], low maternal education has been indicated as a key risk factor for breastfeeding cessation. In contrast, another study found that the age, academic qualifications or parity of the mother were not associated with different rates of MOM feeding at discharge [66].

In a large cohort [92] from 124 NICUs in the United States, an older maternal age, white race, greater gestational age and the site of care were significant predictors of increased MOM use at discharge. In California, the absence or shortage of prenatal care, a young maternal age, and Hispanic and African American heritage were associated with higher rates of FM feeding at discharge for VLBWs [93].

In another series [84], younger and less educated mothers were more likely to stop breastfeeding before 6 months. In addition, other factors such as multiple birth, BPD and neonatal transfers impacted the probability of MOM feeding continuation. Among them, two neonatal factors negatively influenced MOM feeding maintenance: BPD and neonatal transfers [94]. 

In a report [95] in which the association of MOM feeding with natural pregnancies or assisted fertilization was studied, the factors associated with the early cessation of MOM feeding were smoking during pregnancy, birth weight ≥ 1000 g, gestational age ≥ 29 weeks, single-mother status, a short (<12 years) duration of maternal or paternal school education and natural conception. 

### 4.2. Modifiable Factors

It is easy to understand that the frailty, vulnerability and limited neurological competence of preterm infants could compromise MOM use and breastfeeding in a neonatal intensive care unit, but other modifiable factors have been well described. These are, for instance, mother/baby separation, the anxiety and stress accompanying the birth of a high-risk baby, the mother’s difficulty expressing milk and the potentially suboptimal performance of the NICU staff [96]. 

Hospitalization in an NICU and the separation of the mother–child dyad are significantly associated with reduced rates for the initiation and frequency of breastfeeding [97,98]. Also, previous breastfeeding experiences have protective effects on breastfeeding in the NICU. In particular, it was found that women who have not previously breastfed are 5.6 times more likely to stop exclusive breastfeeding before discharge than those who have previously breastfed for at least 4 months [53].

The use of MOM at discharge is associated with higher rates of continued breastfeeding in the following months. Premature infants discharged with breast milk and formula were half as likely to be breastfed at 6 months compared to those who received only breast milk at discharge [99].

The first week is important for the success of feeding with MOM. This is confirmed by a study attesting that, in babies born between 23 and 31 weeks of gestation, a high intake of breast milk during the first postnatal week is associated with higher rates of nutrition with exclusive MOM at 36 weeks [68].

The reception of MOM by day 3 of age was the main predictor of breastfeeding at discharge [62].

It is worth mentioning that it is useful to start trophic feeding with breast milk, improving both structural and functional gastrointestinal development in the preterm. The early availability of milk also facilitates the oropharyngeal administration of colostrum, which can reduce clinical sepsis [100]. The early expression of breast milk plays a similar role in the early initiation of breastfeeding in term infants, for the success of exclusive breastfeeding [101]. 

Only 3.3% of mothers of preterm children started breast expression within an hour of delivery in a study conducted in Northern India [102].

A cross-sectional study conducted in Finland revealed that 36% of mothers begin expressing breast milk within six hours of birth [103], while a study conducted in Japan showed that only 17% of mothers start expressing milk within six hours of delivery. 

In our NICU, we try to start minimal enteral feeding with bank milk as soon as possible and, when clinical stability allows it, within the sixth hour of life. Immediately after premature birth, mothers receive the breast pump kit and information about the breast stimulation protocol. This provides the mother with first access to the kit of extraction within 6 h of birth and then every 3 h, in order to obtain adequate breast stimulation that favors the production of colostrum. When breast milk becomes available, DM is replaced by MOM. Daily increases in milk volumes are planned by a dedicated protocol and are adapted according to the clinical condition and the degree of compliance with enteral feeding.

An important role is played by the emotional challenges of mothers after premature birth [104,105]. Acting on conscious motivation is one of the paths that is most destined for success. It has been shown that the active involvement of the mothers of VLBWs, through the transmission of information on the benefits of using MOM for premature babies and advice on milk extraction and breastfeeding practices, is not a cause of additional stress. Indeed, it is a simple and easily achievable way to make these women co-protagonists of the care of their children, in every moment of life in the NICU, even in the most critical situations [106,107].

Infants with valid direct breastfeeding at discharge are breastfed longer than those receiving feeding bottles [108,109]. 

Research also reports that mothers with insufficient breastfeeding in the NICU experience many more breastfeeding-related challenges after discharge [110,111,112]. For this reason, all methods that have proven their effectiveness in promoting breastfeeding initiation in the NICU should be recognized, enhanced and standardized [86,113].

A study [102] conducted in Shanghai showed that the objective of prolonged exclusive breastfeeding is affected by many factors acting at the individual, family and social levels, and that targeted intervention measures should focus on these three levels.

According to the literature, NICU practices are largely responsible for premature infants’ lack of breast milk intake and/or the early cessation of exclusive breastfeeding [103].

A lack of effective communication, counseling and breastfeeding support contributes to a significant delay in the availability of MOM [110].

Exclusive nutrition with MOM has increased in NICUs with dedicated spaces and breastfeeding support staff [48,111].

In fact, effective interventions to promote breastfeeding and HM use in neonatal intensive care are well known, although they are applied inconsistently: (1) free access of parents to the NICU [12,13], (2) adequate knowledge of the topic of breastfeeding, (3) peer support at the hospital, (4) promotion of breast feeding and assistance to mothers during Kangaroo Mother Care (KMC), and (5) a clear plan encouraging breast milk expression, accompanied by the active promotion of this practice [69].

KMC is a comprehensive intervention; it is suitable and useful, as well as being the most feasible and preferred intervention used to reduce neonatal morbidity and mortality. It is the most effective way to promote the early onset of breastfeeding [112]. 

Systematic reviews and meta-analyses have shown that the KMC certainly has positive effects on growth and breastfeeding rates in VLBW [86,114] infants. Therefore, KMC for preterm and low-birth-weight infants must be systematically promoted and supported by all health facilities that welcome them. 

The main challenges associated with supporting breastfeeding in the NICU were the lack of facilities able to support the opening of NICUs to parents, barriers to breast milk expression and administration, and a high FM feeding rate. Long-distance commuting to the NICU adversely affected mothers’ proximity to their babies and also breast milk extraction and transport frequency [66].

In our NICU, there is an adjacent room for the accommodation of mothers of premature babies, as well as a dedicated place for milk extraction, and this factor has been proven to be one of the most elements that has the greatest impact on the possibility of feeding VLBWs with MOM. 

Mothers’ intention to breastfeed had a significant impact on the duration of milk expression and breastfeeding [115]. 

Mothers and families of children in NICUs should receive both integrated psychological/motivational and practical support.

The partner’s support in the supply of breast milk also promotes the mother’s motivation [66]. 

A qualitative study [116] suggests how fathers can support the MOM feeding of a premature infant. Caregiver intervention in the first days of life through targeted information and practical advice can help fathers to get involved in this process.

In a study [117] carried out in a Kangaroo Mother Care Unit, it was confirmed that this practice increased the direct breastfeeding rates of preterm infants and its efficacy, and had a positive influence on mothers’ intention to continue breastfeeding following discharge and to breastfeed exclusively for six months. The importance of the NICU staff and KMC unit’s role in mothers’ readiness and confidence to breastfeed beyond discharge was emphasized.

We report a very useful consideration of a recent study [76], which recognizes in the NICU an environment well suited to a policy of breastfeeding education and support for mothers, considering the long periods of hospitalization and presence. In particular, the NICU is the most appropriate location for educational practices, as mothers are in close contact with health caregivers. It is also said that health professionals should identify mothers at high risk of the early cessation of breastfeeding, and dedicate supportive interventions to reducing the barriers that prevent this subpopulation of mothers from feeding their premature babies with MOM. All NICUs, as a priority, should have established procedures for breastfeeding protection and support, and practical/organizational methods to facilitate the expression and transport of breast milk. The family-centered NICU has been a main focus of care promoted by all neonatal scientific societies. 

DM availability [118] is considered as another effective element in promoting feeding with MOM, also through an earlier start to enteral feeding in VLBWs.

These findings are summarized in Table 2. 

## 5. Human Donor Milk Use in NICU 

There is no unanimous opinion on the most appropriate time to start the enteral feeding of preterm infants, and this choice is generally individualized, according to their clinical condition. It is recommended that it happens within the first 6 h and no later than 48–72 h. In addition, the advantages of early Minimal Enteral Feeding (MEF, up to 20 mL/kg/day in the first week of life) in VLBWs and ELBWs are well described.

In fact, it is believed that Minimal Enteral Feeding exerts a positive effect on the development of intestinal function and on food tolerance. In particular, it determines a trophic effect on the intestinal mucosa, influences the motor migrating activity due to the effect on the intestinal muscle component, determines a postprandial decrease in the resistance of the splanchnic vascular bed and an increase in intestinal blood flow, with consequent increased oxygen uptake. 

It has been shown [119] that preterm babies requiring parenteral nutrition who are fed with MEF reach Full Enteral Feeding (FEF) 2.7 days before subjects who are not enterally fed, with their hospital stay reduced, on average, by 15.6 days. Until MOM becomes available, DM should be used, as recommended by WHO [120]. Despite the impact of treatments on the bioactive components of breast milk, many positive effects persist. Moreover, the HTST (High Temperature Short Time) method, which is much more conservative of human milk components, is already operational [121].

Human Milk Banks (HMBs) provide for the collection, treatment and distribution of donated human milk, ensuring its quality and safety and acting as a bridge between donors and recipients. Human milk banking should be an integrated component of care in NICUs and a key tool for supporting milk extraction and breastfeeding [121]. All NICUs should at least be able to provide donated human milk in an integrated supply system. 

**Figure 1:**
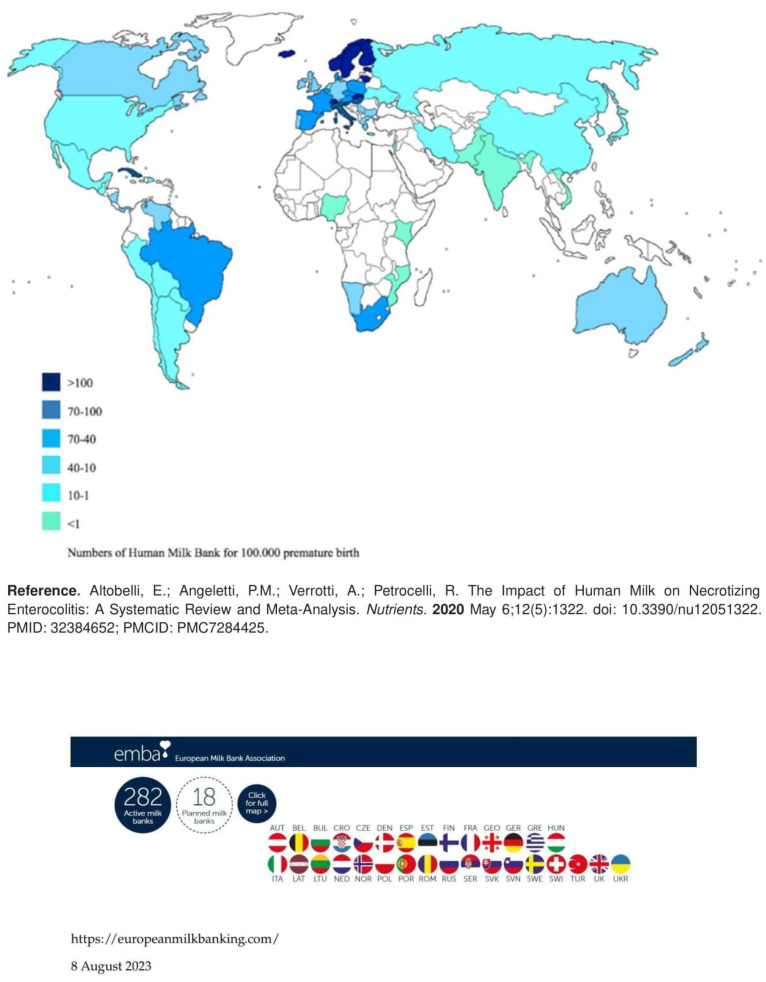
Human milk banking in the world [6].

The number of HMBs around the world is constantly increasing (Figure 1). However, it is believed that, with an estimate of 15 million premature babies born each year globally and given the importance of human milk for this category of newborns, there is a pressing need to increase human milk banking worldwide [122,123]. 

In 2020, the HMB world map [124] identified 756 HMBs in 66 countries, feeding 800,000 newborns each year; Brazil was a leading country, with a network of more than 200 HMBs. Also, in low- and middle-income countries, a growing number of milk banks were established. 

The authors of a study conducted in 2021 [125] reported that only 66% of Level 3 neonatal intensive care units and 73% of Level 4 NICUs use DM in the United States. However, in many countries, the availability of human milk donated in the NICU does not even cover 20% of the needs.

In a recent study [126] in Brazil, a remarkable growth in breastfeeding support has been reported over the last decade, with a 49% increase of group support and a 62% increase of individual support provided through the Brazilian network of HMBs. The number of the latter, in turn, grew by 52% along with milk collection stations. In addition to these data reported by Carrijo et al., there is evidence of substantial growth of HMBs in other geographical areas [127]. Between 2010 and 2019, the North American Human Milk Banks Association (HMBANA) experienced an almost 400% increase in DM volume, and the number of milk banks tripled [128].

In Guatemala, eight milk banks were opened between 2008 and 2017, thanks to Brazil’s support. Between 2013 and 2016, China opened its first 14 HMBs and the number of donors increased from 309 in 2013 to 1429 in 2016; in the same years, the total number of donations increased from 1273 to 8331 and the volumes of DM grew from 230.9 to 2740.7 L [129]. 

In 2016, 11 HMBs were founded in Iran, and the first milk banks were also opened in Vietnam, Kenya, Uganda and Croatia [130,131,132]. 

However, there are still many inequalities in the milk banking system, particularly in low- and middle-income countries, and in regions such as sub-Saharan Africa, where demand exceeds availability and newborns are 10 times more likely to die than children in high-income countries [133].

As rightly stated in one study, the capacity to provide human milk to all infants who need it reflects a country’s ability to achieve important health and development goals, caring for neonatal health, complying with minimum quality and safety standards, and using efficient quality management systems [134]. 

Inter-state agreements and international conventions should be actively promoted and enacted in the field of lactation care, in order to issue minimum standards of quality, safety and ethics that are valid for all milk banks in the world. The basic processes must be valid in all local contexts, even in the presence of differences that may concern the needs or resources of each territory, as well as cultural, religious and traditional characteristics. All HMBs share many common challenges and should cooperate and coordinate their actions through international partnerships in order to be able to apply the same set of quality standards and thus improve the care of their most fragile patients on a global scale [135].

This issue has become more evident during the COVID-19 pandemic, where human milk banks experienced an increase in demand and a dramatic drop in donations. However, this pandemic also stimulated collaboration and sharing between milk banks around the world. In the most dramatic period, a virtual collaborative network was activated, with the commitment of the different groups involved, to form the Global Alliance of Milk Banks and Associations (GAMBA) [136].

This need is evident in the increasingly frequent occurrence of natural disasters and civil conflicts that create humanitarian crises, during which providing breastfeeding support to mothers and newborns becomes difficult, despite it being fundamental to cope with the severe risks associated with these serious situations. Having a comprehensive set of rules and policies, based on well-documented evidence, can play a crucial role in preparing the local health service to protect breastfeeding during difficult periods and, as a result, to save many lives. More than ever, there is an urgent need to raise awareness of the benefits of using maternal and donated milk in such tragic situations. 

In Europe, there are 282 active human milk banks, with France, Italy and Sweden having the largest number of banks. The EMBA (https://europeanmilkbanking.com/) (accessed on 2 July 2023), a non-profit organization that promotes human milk banking and encourages international cooperation between HMBs of European countries, has also been established (Figure 1).

In Italy, there are currently 41 structures regulated by the Ministry of Health and coordinated by the Italian Association of Milk Banks (AIBLUD), which has published guidelines on the creation and mode of operation of human milk banks. However, Italian HMBs meet the needs of only 26% of VLBWs, with some regions covering 100% and others less than 10%. That is why regional networking projects of all Italian milk banks are underway; this is based on the model of Brazil, which has successfully demonstrated the effectiveness of a nationalized human milk banking program in which there are integrated breastfeeding support centers with donated milk collection stations, with the ultimate goal of ensuring equal access to human milk for all babies. 

### Concluding Remarks

**Figure 2:**
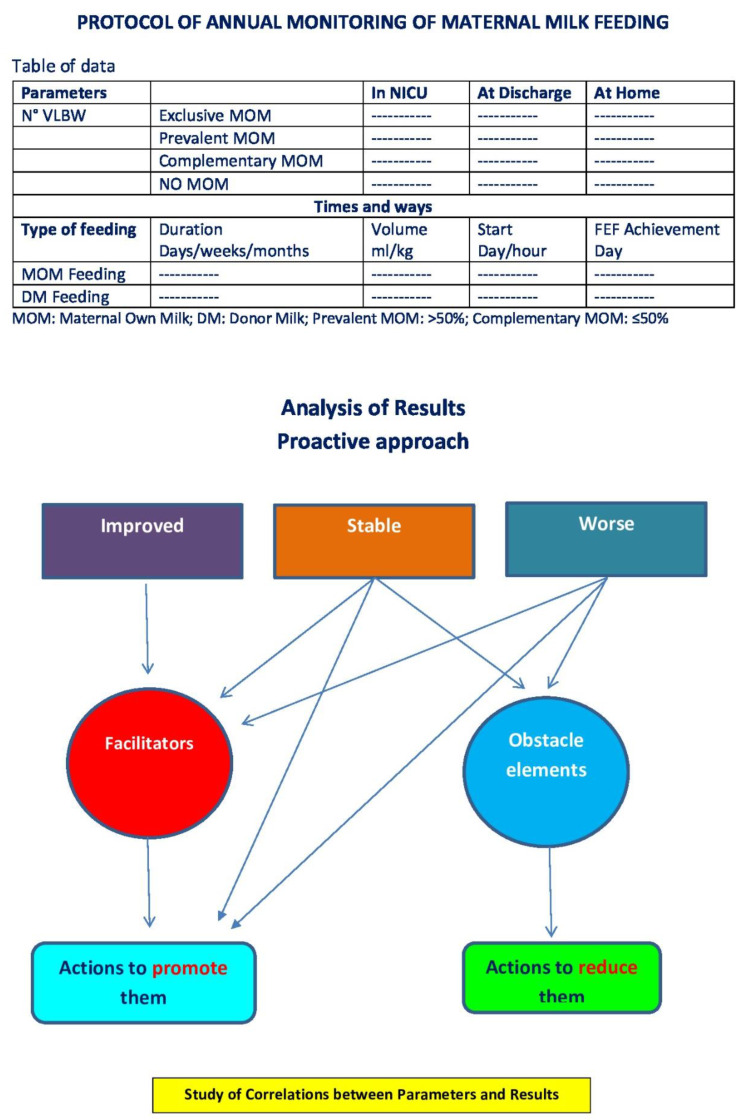
Proposed monitoring and action protocol.

**Figure 3:**
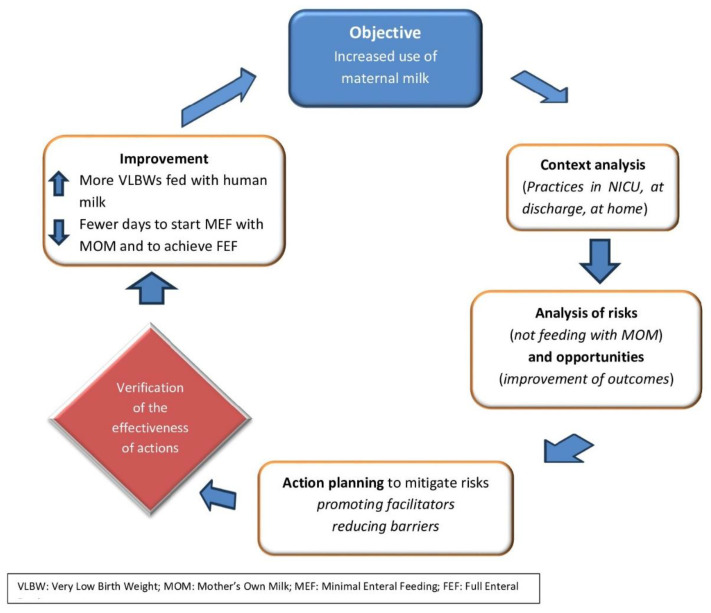
Objective–analysis–improvement loop.

Overall, systematic monitoring data on the feeding of VLBWs with breast milk are lacking. This information is necessary so that practices that favor the achievement of the objectives indicated by health institutions can be embraced. Since the maximum protection induced by breast milk is achieved when vulnerable infants receive high doses and long exposure to breast milk, the indicators to be known are as follows: the extent of nutrition with MOM during hospitalization, at discharge and in the following months, the timing of enteral feeding initiation, the achievement of full enteral feeding, and the amount of human milk consumed (Figure 2 and Figure 3). It would also be desirable to study and to systematize, through multicenter and international analysis, the factors that may affect the success of MOM feeding and breastfeeding in this category of infants most at risk.

Unpredicted events such as the COVID-19 pandemic strongly impacted nutrition with MOM, and all states should already have measures in place to combat this negative effect. It has also made us aware of the importance of a more systematic data collection process. In order to analyze gaps in lactation care, it is necessary to assess the human milk feeding practices in NICUs and at home. Without the proper measurement of data, there can be no verifiable change in practice. 

Even with the few data currently available, the promotion measures put in place by many governments are enhancing the feeding of VLBWs with human milk, although such a result is not yet sufficient. 

**What is Known**

The intake of maternal milk in preterm infants has the ability to significantly mitigate the effects of premature birth by preventing neonatal morbidities, such as NEC, infections, ROP, and BPD, which makes this aspect a crucial element of care in NICUs.

The impact is stronger if the volume of MOM consumed is greater and the exposure to MOM is longer.

The effects of MOM use on the health of this category of newborns produce significant cost savings in the short and long term.

**What is New**

Data on the volume and time of the exposure of very low-birth-weight infants to their mother’s milk and, in cases where it is not available, to donated milk, are not collected systematically.

Attempts to understand the factors that act as facilitators or obstacles to feeding babies with their mother’s own milk are not exhaustive.

Globally, we can say that signs of an increase over time in feeding with MOM and breastfeeding rates can be observed, but there is still ample room for improvement.

There is a discrepancy between the well-known methods of breastfeeding promotion and their application.

The absence of the systematic monitoring of this element of premature baby care may invalidate some of the results presented in this review. 

## Figures and Tables

**Table 2:** Factors affecting MOM use.

FACTORS	EFFECTS		
	Less MOM	More MOM	References
**Non-Modifiable Factors**			
Maternal Age	Younger		[51,66,78,79,80,84,92]
Gestational Age	Lower		[53,62,78,79,92]
Birth weight	Lower		[78]
Parity	Multiparous		[51,78,79,80]
	No effect		[66]
Ethnicity and race Hispanic African	Lower		[92,93]
Multiple Birth	X		[53,78,84]
		X	[44]
Marital status	Not married		[51,78,79,80,89,90,95]
		Married	[44]
Educational status	Lower		[51,54,78,79,80,84,91,95]
Morbidities	Severe		[78,79,94]
Type of Conception	Natural		[95]
**Modifiable Factors**			
Smoke	X		[51,78,79,80,95]
Prenatal Care	Poor or absent		[51,78,79,80,82]
Maternal/neonatal unit policy	Dyad separation		[67,78,80,81,82,83,96,97,98]
	Stress, difficulty in expressing milk		[96,104,105]
	Long hospitalization		[78,79,97,98]
		MOM at discharge	[99]
	Lack of communication, counseling Lack of breastfeeding support		[110,111]
	Lack of mother’s proximity		[66]
		Dedicated spaces	[48,111]
		Free access of parents	[12,13]
		MOM within day 3 of age	[62]
		High intake of MOM during the first postnatal week	[68]
		Breastfeeding at discharge	[108,109]
		Availability of DM	[67,118]
		Active breastfeeding promotion/support of staff	[51,69,76,78,80,81,82,83]
		Baby-Friendly Hospital accreditation	[67,76]
		Support of partner and family	[66,116]
		KMC	[51,112,113,114,117]
Previous breastfeeding experience	No previous breastfeeding (5–6 times more likely to stop before discharge)		[53]

## Data Availability

No new data were created or analyzed in this study. Data sharing is not applicable to this article.

## Abbreviations

BPD	Bronchopulmonary Dysplasia
DM	Donor Milk
EF	Enteral Feeding
ELBW	Extremely Low Birth Weight
EUGR	ExtraUterine Growth Retardation
FEF	Full Enteral Feeding
FM	Formulated Milk
GA	Gestational Age
HM	Human Milk
HMB	Human Milk Bank
LOS	Late-Onset Sepsis
MEF	Minimal Enteral Feeding
MOM	Mother’s Own Milk
NEC	Necrotizing Enterocolitis
NICU	Neonatal Intensive Care Unit
RCT	Randomized Controlled Trial
ROP	Retinopathy of Prematurity
VLBW	Very Low Birth Weight
VLBWs	Very Low-Birth-Weight infants
